# The Size and Localization of Ribeye and GluR2 in the Auditory Inner Hair Cell Synapse of C57BL/6 Mice Are Affected by Short-Pulse Corticosterone in a Sex-Dependent Manner

**DOI:** 10.3390/brainsci15050441

**Published:** 2025-04-24

**Authors:** Ewa Domarecka, Heidi Olze, Agnieszka J. Szczepek

**Affiliations:** 1 Department of Otorhinolaryngology, Head and Neck Surgery, Charité-Universitätsmedizin Berlin, Corporate Member of Freie Universität Berlin and Humboldt-Universität zu Berlin, 10117 Berlin, Germany; ewa.domarecka@charite.de (E.D.); heidi.olze@charite.de (H.O.); 2 Faculty of Medicine and Health Sciences, University of Zielona Góra, 65-046 Zielona Góra, Poland

**Keywords:** cochlea, inner hair cell synapse, AMPA, glutamate receptor subunits, stress hormones, corticosterone

## Abstract

**Background:** Inner hair cell (IHC) ribbon synapses are the initial synapses in the auditory pathway, comprising presynaptic ribbons and postsynaptic glutamate receptors on the peripheral afferent fibers. The excitatory neurotransmitter glutamate primarily signals through AMPA-type heterotetrameric receptors (AMPARs), composed of GluR1, GluR2, GluR3, and GluR4 subunits. Research shows that corticosterone affects AMPA receptor subunits in the central nervous system. The present study investigates the effects of corticosterone on AMPA receptor subunits in the murine cochlea. **Methods**: Cochlear explants were isolated from male and female C57BL/6 pups (postnatal days 4–5), treated for 20 min with 100 nM corticosterone, and cultured for an additional 24 h. The concentration of AMPAR protein subunits was quantified using an ELISA assay, while gene expression was analyzed using RT-PCR. The synaptic localization patterns of GluR2 and Ribeye were examined using immunofluorescence and confocal microscopy. **Results**: Male C57BL/6 mice have a significantly greater basal concentration of the GluR2 subunit than females and more GluR2 puncta per IHC than females. Corticosterone increases the size of Ribeye in males and increases twofold GluR2/Ribeye colocalization in the apical region of females. **Conclusions**: Exposure of membranous cochleae to corticosterone induces changes consistent with neuroplasticity in the auditory periphery. The observed effect is sex-dependent.

## 1. Introduction

The fast-acting neurotransmitter glutamate and its receptors are crucial in transmitting signals from the inner ear to the auditory cortex. A critical event in the process of auditory signal transduction is the activation of spiral ganglion neurons (SGNs). The initiation of this process is marked by the release of glutamate from the ribbon synapses of inner hair cells (IHCs) [1]. After this initial interaction, glutamate binds to the AMPA receptors located on the postsynaptic membrane of the SGNs, thereby inducing a series of physiological responses. These include sodium influx, depolarization, and generation of an action potential. The number of afferent synapses on the IHCs is tightly regulated and varies along the tonotopic axis of the cochlea, reflecting auditory sensitivity in a dose-dependent manner [2]. Understanding the changes in synaptic morphology and the conditions that lead to such changes is essential for understanding their role in auditory health and disease [3].

Stress is defined as an adaptive response of an organism to change. The physiological response to stress in humans and animals involves activating the hypothalamic–pituitary–adrenal (HPA) axis, which triggers the release of corticosteroids by the adrenal glands [4]. In humans, the primary corticosteroid released in response to stress is cortisol, whereas in rodents, the primary corticosteroid released is corticosterone (CORT). Numerous basic and clinical neuroscience studies have used animal models to investigate the physiological effects and consequences of stress, with a particular focus on acute and chronic exposure to stress hormones such as CORT [5].

CORT has been shown to elicit varied effects on the heterotetrameric α-amino-3-hydroxy-5-methyl-4-isoxazolepropionic acid receptors (AMPARs), which consist of glutamate ionotropic receptor AMPA-type subunits GluR1, GluR2, GluR3, and GluR4 encoded by the respective genes Gria1–4 (1–4). Acute stress impacts the glutamate transmission system through glucocorticoids [6]. These effects include changes in AMPA subunit transcription and non-genomic alterations in AMPA synaptic trafficking [7]. The transcriptional regulation of AMPA subunits varies depending on the brain area, the type of exposure (single or repeated), and the duration of exposure to CORT [7,8,9]. Non-genomic alterations were observed in neurons derived from the embryonic hippocampal tissues of E18 Sprague Dawley rats, where increased GluR2 surface trafficking was noted after 10 min of exposure to 100 nM corticosterone [7]. In a similar experimental model, the surface expression of GluR1 and GluR2 increased after three hours of exposure to 30 and 100 nM corticosterone, with more pronounced changes in GluR2 [10]. In a recent scoping review, we investigated the effect of corticosterone on AMPA receptors in the central nervous system (CNS) of rodents [11]. We summarized the impact of corticosterone on AMPAR levels and localization in the rodent nervous system, as reported in 17 studies, and concluded that brief exposure of hippocampal neurons to corticosterone increases the quantity of GluR2 subunits in the postsynaptic plate. In contrast, long-term exposure to corticosterone, which is associated with chronic stress, has been shown to reduce the number of GluR2 subunits [12]. These structural changes may promote synaptic plasticity mediated by AMPA receptors.

A recent study has uncovered sex-based variations in the sensitivity of AMPA receptor subunits within the dorsal hippocampus. Restraint stress for two hours resulted in alterations dependent on sex, time, and memory in adult C57BL6/J mice. While changes were noted in both genders, a notable increase in the mRNA expression of Gria1 and Gria2 following stress was observed solely in female mice. These stress-induced changes may stem from distinct sex-dependent molecular mechanisms, although differences in individual stress responses have also been documented [13]. Besides the variations in synaptic glutamate signaling between sexes, sex also impacted the baseline composition of AMPA receptors [14,15].

The direct impact of emotional stress and stress hormones, such as cortisol or corticosterone, on the auditory system, particularly the cochlea, remains poorly understood. Basic research studies have demonstrated that 24 h moderate stress can alter the hearing threshold in female rats, as indicated by DPOAE and ABR [16], and these changes depend on the animal strain [16]. Also, experimental stress has been shown to induce an auditory threshold shift in guinea pigs [17]. Nevertheless, the underlying mechanisms of auditory modulation following stress have yet to be examined.

Based on existing clinical and basic research data, we hypothesized that stress hormones may directly affect the synapse between IHCs and SGNs. Therefore, our study aimed to determine the impact of brief corticosterone exposure on gene and protein expression and the localization of AMPA subunits in the IHC synapse of murine cochleae.

## 2. Materials and Methods

### 2.1. Ethical Statement and Animals

The experiments were approved by the Governmental Ethics Commission for Animal Welfare (LaGeSo Berlin, Germany; approval numbers: T 0292/16 and T-CH 0036/22). The animals were sacrificed to obtain tissues immediately after transfer from the animal facility. C57BL/6 mice (P4–P5, both sexes) were purchased from a local animal facility. The day of birth was defined as postnatal day 0 (P0). The cochlear explants were randomly assigned to control and CORT-exposed groups.

### 2.2. Experimental Flow

**Figure 1:**
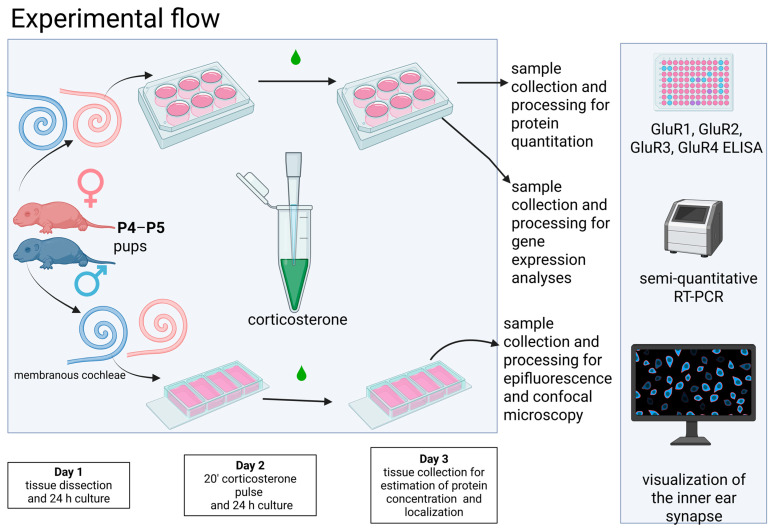
Cochlear tissues were dissected from postnatal day (P4–P5) male and female pups and placed into culture for 24 h. Subsequently, the tissues were subjected to a 20 min pulse exposure to 100 nM CORT, followed by three washes and a 24 h incubation period. Next, protein concentration was analyzed using ELISA, and relative gene expression was determined by semiquantitative real-time RT-PCR. Protein localization was visualized using confocal microscopy. The manuscript uses pink to represent female data and blue for male data. Created with Biorender.com.

The study adheres to the following experimental protocol: cochlear tissues were dissected from postnatal day (P4–P5) male and female pups and subsequently placed into culture for 24 h (Figure 1). Following this, the tissues underwent a 20-min pulse exposure to 100 nM CORT, which was followed by three washing cycles and a subsequent incubation period of 24 h. Three techniques were employed: ELISA, real-time RT-PCR, and confocal microscopy. ELISA assessed the concentration of each AMPAR subunit, semiquantitative real-time RT-PCR was employed to evaluate relative gene expression, and confocal microscopy was utilized for protein localization and quantification. Each specific experimental procedure is detailed in the following Materials and Methods subsections.

### 2.3. Cochlear Explant Culture

Murine cochleae were dissected from temporal bones in cold DMEM medium (DMEM/F12 (1X), cat. no. 21041-025, Thermo Fisher Scientific, Dreieich, Germany) following a previously described protocol [18]. The membranous part of the cochlea, containing the organ of Corti, spiral limbus, and spiral ganglion neurons, was carefully separated from the stria vascularis, spiral ligament, and tectorial membrane using Dumont #5 fine forceps (cat. no. 11254-20, Dumont, Montignez, Switzerland). After that, the cochlear tissues were explanted onto Millicell EZ SLIDE 4-well glass (cat. no. PEZGSO416, Merck Millipore, Dublin, Ireland). In preparation for ELISA and RT-PCR, the explants were incubated in a 4-well Nunc™ plate (cat. no. 176740, Thermo Fisher Scientific, Dreieich, Germany). Each well contained 500 µL of pre-warmed tissue culture medium (DMEM/F12, cat. no. 21331020, Thermo Fisher Scientific, Dreieich, Germany) supplemented with 10% heat-inactivated Fetal Bovine Serum (FBS, cat. no. 10500-64, Thermo Fisher Scientific, Dreieich, Germany), 2.5 M D-(+)-glucose solution (cat. no. G8769, Sigma-Aldrich, Darmstadt, Germany), penicillin G sodium salt (cat. no. A321-42, Biochrom AG, Berlin, Germany), insulin-transferrin-Na selenite supplement (cat. no. 1074547, Roche, Basel, Switzerland), and the recombinant mouse IGF-1 (cat. no. 4326, Bio-Techne GmbH, Wiesbaden, Germany). All explants were cultured at +37 °C and 5% CO_2_ for 24 h before performing experimental procedures.

### 2.4. Exposure to Corticosterone

A short-pulse CORT exposure model, developed to study stress-induced changes in organotypic cultures of the mouse brain [19], was adapted for cochlear explant cultures. After 24 h preincubation, cochlear explants were exposed for 20 min to 100 nM CORT. To exclude the effect of diurnal variation in systemic CORT concentration, animals were sacrificed between 9:00 and 10:00 when plasma CORT levels in mice were low [19,20]. A stock solution of CORT (cat. no. 27840, Sigma-Aldrich, Darmstadt, Germany) was prepared by dissolving corticosterone in DMSO (cat. no. 20385.01, Serva, Heidelberg, Germany) to a concentration of 100 mM and then in ethanol (cat. no. E/0600DF/C17, Fisher Scientific, Schwerte, Germany) to a final concentration of 10 mM. Next, the stock solution was diluted 1:1000 in a freshly prepared tissue culture medium and added 1:100 to the tissue cultures. After a 20 min incubation at +37 °C and 5% CO_2_, the cochlear explants were gently rinsed three times with warm tissue culture medium and cultured for an additional 24 h without CORT. The tissues were either fixed for immunofluorescence, lysed for ELISA, or homogenized for RNA extraction, as described later. Control tissues were maintained under the same conditions with just a culture medium.

### 2.5. Quantification of the AMPA Receptor Subunit Concentrations in the Cochlear Tissues by Enzyme-Linked Immunosorbent Assay (ELISA)

Commercially available ELISA kits were used to measure the concentration of the glutamate receptors in the cochlear explants (Table 1). Cochleae were collected and lysed, and the total protein concentration in the lysate was estimated using the micro-BCA protein assay (cat. no. 23235, Life Technologies GmbH, Darmstadt, Germany). Next, the samples were diluted 1:26 with DPBS (DPBS (1x), cat. no. 14190-094, Thermo Fisher Scientific, Dreieich, Germany) to obtain 800 µL of solution and assayed in duplicates according to the manufacturer’s instructions. The optical density of samples (after adding the substrate) was measured at 450 nm using Spectramax M2 (Molecular Devices, Sunnyvale, CA, USA). The results were computed with SoftMax Pro, V5 software (Molecular Devices).

### 2.6. Treatment of Cochlear Explants with Glucocorticoid Receptor Antagonists

To identify the glucocorticoid receptor involved in cochlear explant function, we treated the explants with selective antagonists targeting either mineralocorticoid (MR) or glucocorticoid (GR) receptors.

A selective MR antagonist, spironolactone (cat. no. S3378, Sigma-Aldrich, Darmstadt, Germany), was dissolved in DMSO (cat. no. 20385.01, Serva, Heidelberg, Germany) to prepare a stock solution at a concentration of 0.1 mM. On the experimental day, the working solution (100 nM) was prepared in two steps: first, the stock was diluted in freshly prepared tissue culture medium to 10 µM, then to the final concentration of 100 nM.

A stock solution of the selective GR antagonist mifepristone (RU38486, cat. no. A10591, Adooq Bioscience, Irvine, CA, USA) was prepared by dissolving it in distilled water to a concentration of 100 µM, followed by dilution to 500 nM using freshly prepared tissue medium. The solutions with antagonists were freshly prepared 10 min before each application. Both antagonists were applied for 20 min before and during the 20 min CORT incubation, as described previously for brain tissues [21]. After that, the cochlear explants were rinsed with warm tissue culture medium, followed by 24 h of culture in tissue culture medium. Next, the cochleae were collected for ELISA or immunofluorescence to detect GluR2 and Ribeye.

### 2.7. RNA Extraction and Semiquantitative Real-Time One-Step Reverse-Transcription–Polymerase Chain Reaction (sq rtRT-PCR)

Cochlear explant cultures were assessed for mRNA expression changes at two experimental time points: 3 and 6 h after CORT exposure. One-step RT-PCR using specific primers assessed the relative levels of AMPA receptor subunits in cochlear tissue. Four cochlear explants were pooled for each RNA sample, which was isolated using an RNeasy Mini Kit (cat. no. 74106, Qiagen, Hilden, Germany), following the manufacturer’s instructions closely. The samples were collected during several independent experiments to ensure data reliability. Two to three biological samples for each experimental condition were assayed in duplicates. The RNA concentration was measured using the NanoDrop One (cat. no. 701-058112, Thermo Fisher Scientific, Dreieich, Germany). The purity of the extracted RNA was evaluated using the A260/A280 ratio, which ranged from 2.0 to 2.2 in all instances. One-step real-time RT-PCR was performed with the QuantiNova™ SYBR Green RT-PCR Kit (cat. no. 208154, Qiagen, Hilden, Germany), using primers targeting Gria2 (Mm_Gria2_1_SG QuantiTect Primer Assay; GeneGlobe ID—QT00140000, Qiagen) and beta-actin as a housekeeping gene (Mm_Actb_1_SG QuantiTect Primer Assay; GeneGlobe ID—QT00095242, Qiagen). Each of the 20 µL reaction samples contained 100 ng of RNA. The QuantiNova™ SYBR Green RT-PCR Kit employs a two-phase hot-start protocol, activating the HotStaRT-Script reverse transcriptase at 50 °C for 10 min. After completing the RT step, the QuantiNova DNA Polymerase was activated at 95 °C for 2 min, followed by 40 cycles of annealing/extension at 60 °C for 30 s and denaturation at 95 °C for 15 s. The final cycle consisted of 95 °C for 10 s, 60 °C for 60 s, and 97 °C for 1 s, followed by a cooling step to 37 °C. Data were acquired using Instrument Software V1.2 (Roche) set up for SybrGreen [22]. To measure relative quantification, the comparative CT method, also known as the ΔΔCT, was used [23].

### 2.8. Visualization of Hair Cells and SGNs

Cochlear explants were fixed in 10% neutral buffered formalin (cat. no. HT5011, Sigma-Aldrich, Darmstadt, Germany) for 40 min at room temperature and then rinsed twice with PBS (each rinse lasting 15 min). After permeabilization with 0.5% Triton X-100/PBS (cat. no. 9002-93-1, Sigma-Aldrich, Darmstadt, Germany), specimens were incubated in a blocking solution (4% *v*/*v* in PBS of goat serum, cat. no. 005-000-121, Jackson ImmunoResearch Europe Ltd., Ely, UK) for 1 h at room temperature within a humidity chamber to prevent non-specific binding sites. Next, explants were incubated overnight at +4 °C with a primary antibody against neurofilament-200 (to label SGNs), diluted in a solution of PBS with 2% NaCl (340 mM), 0.1% Triton X-100, and 5% NGS (all antibodies are listed in Table 2). The following day, specimens were rinsed three times with PBS (15 min per rinse) and incubated for one hour with a secondary antibody (dilution 1:400) at room temperature, followed by treatment with phalloidin (to visualize the F-actin-rich stereocilia of hair cells), diluted 1:1000 in PBS for 40 min at room temperature. After covering the specimens with a mounting solution and coverslips, they were dried overnight at room temperature.

### 2.9. Immunofluorescent Detection of Cochlear Ribbon Synapses

After being fixed for 40 min in 10% formalin and rinsed twice with PBS for 15 min each, the tissue samples were incubated for 1 h in a blocking solution consisting of PBS, 0.5% Triton X-100, and 10% normal goat serum (cat. no. 005-000-121, Jackson ImmunoResearch Laboratories, Inc., West Grove, PA, USA) in a humidified chamber at room temperature to block non-specific binding sites. After blocking, the explants were incubated for 20 h with primary antibodies targeting Ribeye and GluR2 (Table 2), diluted in 5% *v*/*v* goat serum in PBS containing 2% NaCl (340 mM) and 0.1% Triton X-100 in a humidified chamber at room temperature on a rotator. The following day, after three 30 min washes with PBS, the explants were incubated with secondary antibodies diluted in 0.1% Triton X-100 and 5% goat serum in PBS for 3 h at room temperature in a humidified chamber. The hair cells were visualized by a 45 min incubation with Phalloidin-iFluor 594 at room temperature. At the end of the staining procedure, the specimens were coverslipped with Prolong Gold DAPI. The specificity of the anti-GluR2 antibody was previously confirmed in GluA2-null mice [24].

### 2.10. Fluorescence Microscopy of Cochlear Explants

Primary screening of specimens was conducted using an EVOS FL Cell Imaging System (Thermo Fisher Scientific, Karlsruhe, Germany) epifluorescence microscope. Digital images were captured with ×4, ×20, and ×40 objectives. The fluorochromes used included Alexa 488 (excitation–emission spectrum 496–549 nm), Alexa 594 (excitation–emission spectrum 591–614 nm), and DAPI (excitation–emission spectrum 409–464 nm). ImageJ software version 1.48v (https://imagej.net/ij/, accessed on 8 January 2025) facilitated image restructuring and quantitative analysis.

The entire cochlea was photographed using a 4× objective and measured from apex to base to create a cochlear frequency map. Cochlear lengths were estimated for each specimen by measuring the basilar membrane. Subsequently, the cochlear tissues were optically divided into ten sections (1—apex, 10—base). Sections 1–3 defined the apex, 4–7 the medial region, and 8–10 the base.

### 2.11. Confocal Microscopy

To visualize cochlear ribbon synapses, stained cochlear specimens were examined using a Leica SPE confocal microscope with a 63× oil-immersion objective lens (1.4 numerical aperture) and 1.5 digital zoom. The cochlear tissue was divided into ten equal optical sections, starting at the apical end and ending at the base of the basilar membrane. Confocal Z-stacks were consistently collected from the middle of regions 2 through 9. Regions 1 and 10 were excluded from analysis due to physical damage incurred during cochlear dissection. Each stack contained 10 to 17 IHCs, depending on the cochlear location. The images were merged using ImageJ, each containing at least 30 optical sections. The imaging process utilized standard settings (resolution of 1024 × 1024 pixels, speed of 400 Hz (lines per second)). The channel settings for DAPI, Alexa 488, Alexa 595, and Alexa 647 are detailed below: DAPI 7.67%, “Smart Gain” 1250 V, “Smart Offset” 0%; 488 15.41%, “Smart Gain” 964.8 V, “Smart Offset” −0.1%; 594 5.93%, “Smart Gain” 1158 V, “Smart Offset” 0%; 647 19.2%, “Smart Gain” 1024.5 V, “Smart Offset” 1.8%.

### 2.12. Quantification of Auditory Hair Cells and Spiral Ganglion Neurons

Two images from each of the ten cochlear sections were captured using a 40× objective. When the hair cells were not in the same plane, additional scans were captured and merged into a Z-stack using ImageJ. Hair cell counts were assessed over 100 µm, with approximately 80 cells counted per image. Based on the characteristics of the stereocilia (intact array, aberrant array with missing or fused stereocilia, and absence of stereocilia or cuticular plate), they were classified into three morphological groups: intact, damaged, and missing. Micrographs taken with the 40× objective were used to quantify the spiral ganglion neurons (SGNs). Multiple images from various planes were captured and merged into a Z-stack if necessary. Since AMPA receptors can also mediate the signal of outer hair cells (OHCs) [25], both types of SGN (type I and type II) were assessed. The number of type I SGNs was determined by counting the neuronal fibers on a 100 µm scale. In addition, we evaluated type II fibers that turned incorrectly (toward the apex). The fibers were quantified as described elsewhere [26]. The turning error was calculated as the percentage of type II SGNs that were misturned.

### 2.13. Quantitative Analysis of Synapses

The quantitative analysis of Ribeye and GluR2 puncta and their colocalization in IHCs was performed using ImageJ. After adjusting the contrast level in ImageJ to 40 (always the same for all images), the number of Ribeye and GluR2 fluorescent puncta was counted in each IHC. Only puncta larger than 2 pixels were analyzed to prevent overestimation. The total synaptic counts in each Z-stack were divided by the number of IHCs visualized by phalloidin. The number of IHCs in each analyzed cochlear region varied between 10 and 17. In cases where IHCs in the analyzed section were physically damaged during dissection, the region was excluded from the analysis. The brightness level was adjusted using Adobe Photoshop (version 26.1 (www.adobe.com/products/photoshop.html, accessed on 10 January 2025)).

To examine the morphology of synapses (size, height, width, roundness), the confocal images were rotated and transformed into binary images (black and white), and the “Analyze Particle” function in ImageJ was utilized following previously published instructions [25]. The number of colocalized puncta was quantified using ImageJ software (DiAna plugin). The colocalization between puncta was quantified using the object-based approach [27].

### 2.14. Statistical Analyses

Statistical analyses were performed using IBM SPSS Statistics 27 (IBM Deutschland GmbH, Ehningen, Germany). Based on the data distribution, the results are reported as the mean ± SD or median with a 95% confidence interval. Details are included in the figures and their legends. The Kolmogorov–Smirnov test was used to evaluate the normality of data distribution. Statistical significance was assessed using a two-tailed unpaired Student’s *t*-test for samples that followed a normal distribution. The non-parametric Mann–Whitney U test was utilized when the Kolmogorov–Smirnov test indicated a non-normal distribution. In addition, Cohen’s effect size was reported. The Kruskal–Wallis test was conducted to compare more than two independent groups for non-normal data distribution. A correlation analysis used Spearman’s rank correlation coefficient (rho). The plots were created using GraphPad Prism 7.05. Statistical significance is shown in the figure: * *p* < 0.05; ** *p* < 0.01; *** *p* < 0.001.

## 3. Results

### 3.1. Baseline Differences in Cochlear AMPA Receptor Subunit Concentrations Between Male and Female Mice

Using ELISA, we measured the concentration of each AMPA receptor subunit (GluR1–GluR4). A micro-BCA protein assay was used to measure the total protein concentration, yielding an average of 0.4 µg/µL. The values are expressed as the amount of a specific subunit per µg of total protein [ng/µg].

**Figure 2:**
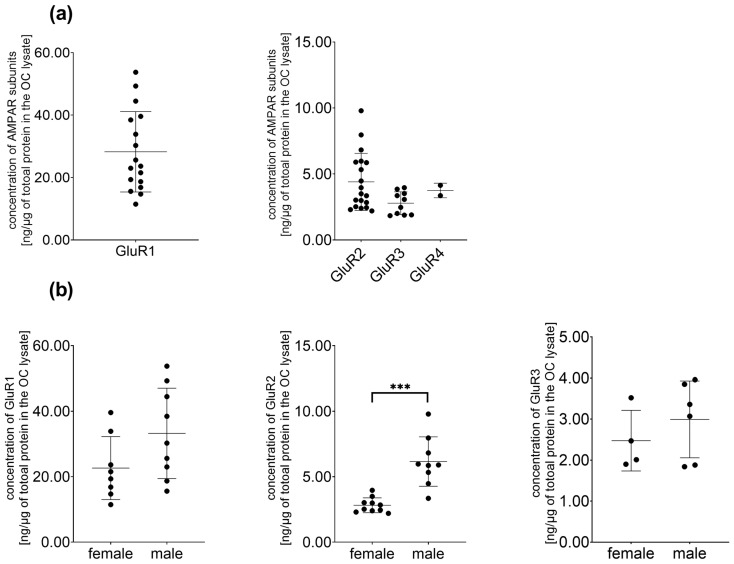
(**a**) Concentration of AMPA receptor subunits in the membranous cochlea of P4–P5 C57BL/6 mice; (**b**) sex influences the baseline concentration of AMPA receptor subunits [ng/µg] in cochlear tissues. The differences in the concentration of GluR1 protein between male and female mice, calculated using *t*-test, were not statistically significant (M: 33.23 ± 13.82, F: 22.62 ± 9.63; t(15) = −1.813, *p* = 0.090, *n* = 8–9 for each experimental group). The concentration of GluR2 was significantly higher in male mice compared to females (F: 2.82 ± 0.57, M: 6.16 ± 1.89, t(9.296) = −5.112, *** *p* < 0.001, Cohen’s d = 2.46, strong effect, *n* = 9–10 cochlear tissues per group). The differences in concentration of GluR3 between males and females were insignificant (M: 2.99 ± 0.94, F: 2.48 ± 0.74, *n* = 4–6 cochlear tissues per group). Mean ± SD is reported. The concentration of GluR4 was below the assay threshold sensitivity in 90% of the samples.

We have confirmed the presence of all four AMPA receptor subunits in cochlear tissues (Figure 2a). A two-tailed unpaired Student’s *t*-test of GluR1 and GluR3 concentrations did not detect significant differences between sexes. However, the concentration of GluR2 was significantly greater in male specimens compared to females (M: 6.16 ± 1.89, F: 2.82 ± 0.57; t(9.296) = −5.112, *p* < 0.001; Cohen’s d = 2.46, strong effect). Moreover, the mean concentration of GluR4 in males was 3.75 ng/1 µg of total protein, but in female specimens, it was below the assay sensitivity limit (Figure 2, Table 3).

### 3.2. CORT Exposure Does Not Affect Cochlear AMPA Receptor Subunit Concentrations

Exposure to CORT did not influence the concentrations of GluR1, GluR2, GluR3, or GluR4 in either males or females (Table 4).

### 3.3. Gria2 Gene Expression Remains Stable 3 and 6 h After the CORT Pulse

Given the baseline differences in GluR2 protein levels between males and females, we sought to investigate whether CORT exposure impacts *Gria2* gene expression. The times 3 and 6 h after CORT exposure were chosen to collect RNA based on the Gria2 expression changes observed in hippocampal tissue slices from Wistar rats after 20 min CORT exposure [28]. One hundred ng of total RNA isolated from the membranous cochleae was subjected to one-step real-time RT-PCR. The RNA isolated from the hippocampus and spleen was used as a positive and negative control, respectively. The results showed no influence of CORT on *Gria2* gene expression in male and female cochleae three or six hours after exposure (Supplementary Table S1).

### 3.4. At Baseline, Male Cochleae Have More GluR2 Puncta than Females and an Equal Number of Ribeye

**Figure 3:**
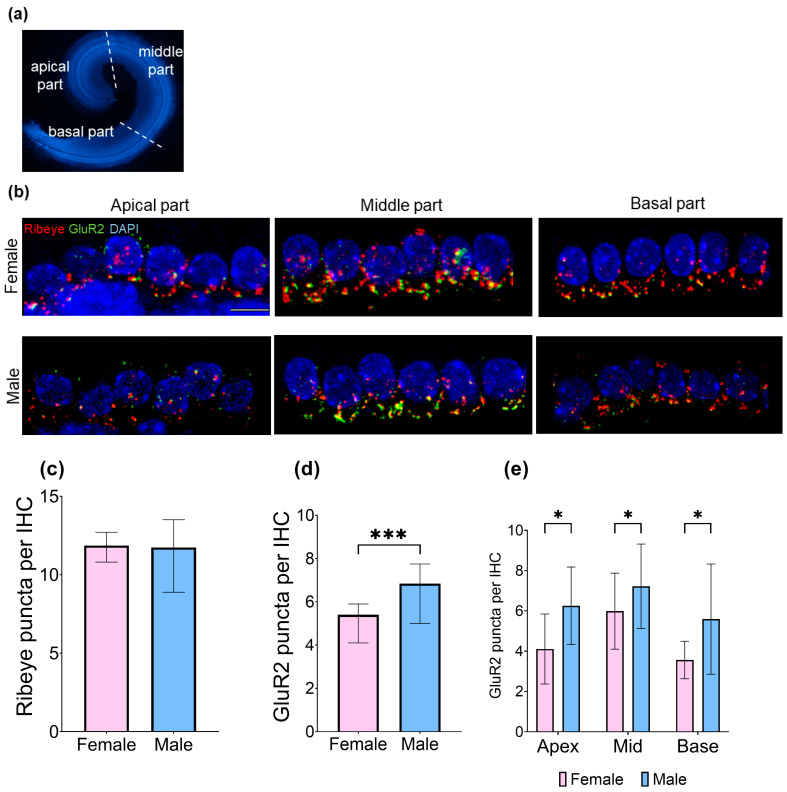
The total number of GluR2 puncta in inner hair cells (IHCs) varies between sexes. (**a**) Overview of the mouse cochlea highlighting the three parts used in further analyses: apical, middle, and basal regions; (**b**) representative confocal images of IHC synapses in the apex, mid, and base areas. The cochleae were labeled with antibodies against Ribeye (red), GluR2 (green), and with DAPI (blue). Scale bar: 10 µm; (**c**) no difference was observed in the numbers of Ribeye puncta per IHC based on sex; (**d**) the number of GluR2 puncta per IHC in the cochlea was significantly higher in male mice compared to female mice (Mann–Whitney U test U = 582, *p* < 0.001, Cohen’s d = 0.35, modest effect). The median ± 95% CI of the median confidence interval is reported; (**e**) a significant difference in GluR2 puncta numbers between male and female animals was observed in the apical part (M: 6.25 ± 1.92, F: 4.11 ± 1.74; t(18) = 2.619, *p* = 0.017, Cohen’s d = 1.17, strong effect), the middle part (M: 7.22 ± 2.10, F: 5.99 ± 1.89; t(43) = 2.077, *p* = 0.044, Cohen’s d = 0.62, moderate effect), and the basal part of the cochleae (M: 5.59 ± 2.74, F: 3.56 ± 0.93; t(13.504) = 2.433, *p* = 0.030, Cohen’s d = 0.99, moderate effect). Mean ± SD is reported, with *n* = 6–8 cochleae for each experimental group, number of analyzed IHCs in total = 1000, average number of analyzed Ribeye puncta per group = 5824, and average number of analyzed GluR2 puncta per group = 2983. Statistical significance is shown in the figure: * *p* < 0.05; *** *p* < 0.001.

Here, we investigated whether there are baseline differences between sexes in the number of presynaptic ribbons (Ribeye) and postsynaptic GluR2 in the inner hair cell synapses. The cochleae were stained with antibodies against GluR2 and Ribeye, and the puncta were quantified. The Mann–Whitney U test demonstrated that the average count of GluR2 puncta per inner hair cell was significantly higher in male specimens (M: 6.56 ± 2.31, F: 4.92 ± 1.97; U = 582, *p* < 0.001, Cohen’s d = 0.35, modest effect). Furthermore, males exhibited a higher number of IHC synapses, as indicated by the percentage of colocalized Ribeye/GluR2 per IHC (M: 30.22 ± 9.21, F: 23.80 ± 9.35; U = 564.5, *p* < 0.001, Cohen’s d = 0.38, modest effect). The number of Ribeye puncta per IHC was unaffected by sex (Figure 3). Dividing the cochleae into three regions—apical (optical parts 2 and 3), middle (optical parts 4, 5, and 6), and basal (optical parts 7, 8, and 9) (Figure 3a)—revealed significant differences in GluR2 puncta counts between male and female subjects (Figure 3d,e).

### 3.5. CORT Exposure Does Not Affect the Number of GluR2 Puncta in Males or Females but Significantly Reduces the Number of Ribeye Puncta in the Middle Cochlear Part and Enhances the Colocalization of Ribeye and GluR2 in the Apical Region of Female Cochleae

**Figure 4:**
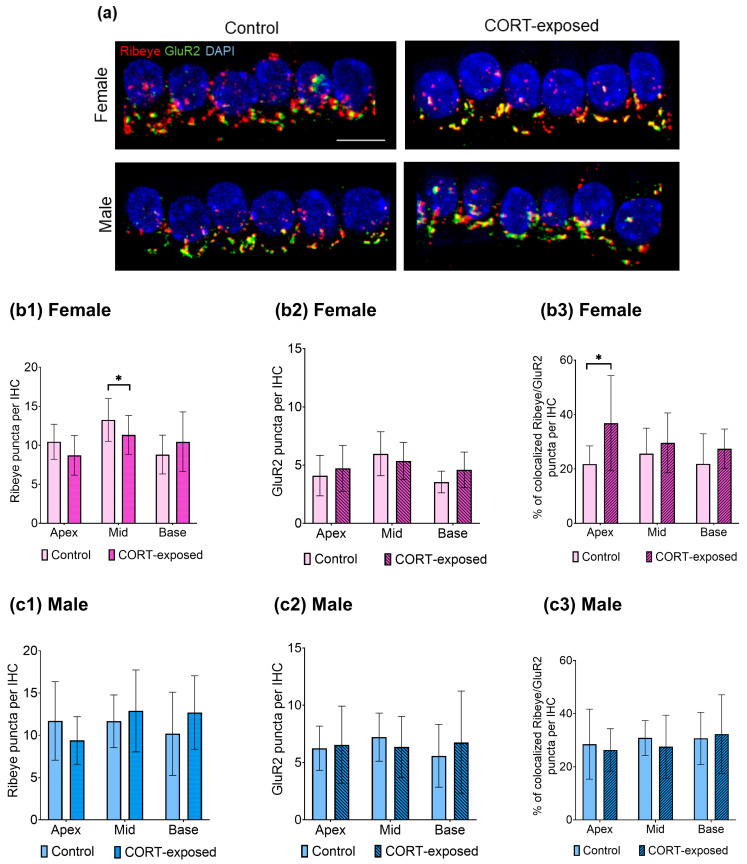
CORT exposure affects the numbers and colocalization of Ribeye and GluR2 puncta in IHCs of female but not male cochlear explants. (**a**) The image depicts the middle part of the cochlea. The explants were cultured in either tissue culture medium alone or exposed to 100 nM CORT for 20 min and cultured for 24 h. The tissues were labeled with anti-Ribeye (red), anti-GluR2 (green), and DAPI (blue), with yellow color indicating the colocalization of the fluorescence signals (Ribeye/GluR2). The scale bar represents 10 µm; (**b1**) reduction of Ribeye puncta number in females after CORT in the middle region of the cochlea (control: 13.26 ± 2.74, CORT: 11.33 ± 2.50; t(45) = −2.532, *p* = 0.015, Cohen’s d= 0.74, moderate effect); (**b2**) no changes in the numbers of GluR2 puncta after CORT; (**b3**) increased colocalization of Ribeye and GluR2 after CORT exposure in the apical region of the cochlea, rising from 22% to 37% (t(14.627) = 2.745, *p* = 0.015, Cohen’s d = 1.10, strong effect); (**c1**–**c3**) CORT does not induced changes in the number of Ribeye and GluR2 puncta or in the colocalization pattern. Mean values are presented ± SD, with *n* = 6–8 cochleae in each experimental group. Total number of analyzed IHCs: 2080; average number of analyzed Ribeye/GluR2 puncta in female group: 6266/2818; average number of analyzed Ribeye/GluR2 puncta in male group: 5880/3297. Statistical significance is shown in the figure: * *p* < 0.05.

We next examined whether the number of Ribeye and GluR2 puncta at inner hair cell (IHC) synapses is altered in CORT-exposed cochlear tissue (Figure 4a). Using an independent samples *t*-test, we found that CORT significantly decreased the number of Ribeye puncta in the female cochlear explants compared to the control group (Figure 4(b1)). This decrease was seen only in the middle region of the cochlea (control: 13.26 ± 2.74, CORT: 11.33 ± 2.50; t(45) = −2.532, *p* = 0.015, Cohen’s d= 0.74, moderate effect). However, no significant changes were observed in male explants after CORT exposure (*p* = 0.323).

Subsequently, we measured the number of colocalized Ribeye and GluR2 puncta in individual IHCs after CORT exposure and observed exclusive changes in the cochlear apex of female subjects. In these samples, the average percentage of colocalized Ribeye/GluR2 puncta increased significantly from 21.82 ± 6.68 to 36.87 ± 17.53 (median values: control, 22%; CORT, 34%) (t(14.627) = 2.745, *p* = 0.015, Cohen’s d = 1.10, strong effect) (Figure 4(b3)).

We then investigated whether the animals’ developmental stage, indicated by body mass, influenced their response to the CORT pulse. The median body mass was 2.8 g for the male group and 2.6 g for the female group (Supplementary Figure S3). Spearman’s rho correlation analysis revealed a significant link between body mass and CORT response, observed only in females. Specifically, the number of Ribeye per IHC (rho = −0.479; *p* < 0.001) correlated with body mass. Additionally, a relationship was noted between female body mass and the quantity of colocalized Ribeye/GluR2 puncta per IHC (rho = −0.559; *p* < 0.001). These correlations demonstrated moderate strength.

**Figure 5:**
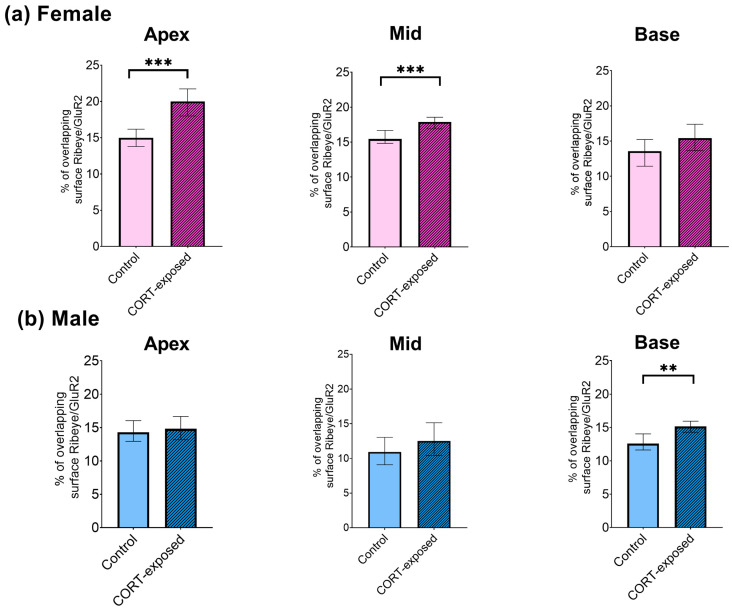
CORT exposure increases the colocalization of Ribeye and GluR2 in cochlear synapses. The changes in the overlapping area of Ribeye and GluR2 were analyzed in control versus CORT-exposed cochleae. (**a**) Female cochlear explants. In CORT-exposed female explants, an increase in the overlapping area was observed at the apex and middle regions. At the same time, no change was seen at the base (apical: 15% to 20%, medial: 15% to 18%, basal: 14% to 15%); (**b**) male cochlear explants. An increase in the overlap between Ribeye and GluR2 was noted in the medial region of the cochlea, rising from 13% to 15%. For all significant changes, Cohen’s effect size was weak (0.1–0.2). The data are presented as median values ± 95% confidence interval (CI) of the median, with sample sizes ranging from *n* = 6 to 8 cochleae for each experimental group. The total number of analyzed IHCs is 2080, with the average number of analyzed Ribeye/GluR2 puncta being 6266/2818 in females and 5880/3297 in males. Statistical significance is shown in the figure: ** *p* < 0.01; *** *p* < 0.001.

Next, we explored how CORT influences the extent of Ribeye/GluR2 surface overlap at IHC synapses in both male and female mice. We used the 3D Distance Analysis plugin (DiAna) to assess the overlapping areas (Ribeye/GluR2) in control and CORT-exposed explants from the cochlea’s apical, medial, and basal regions. CORT exposure significantly increased the overlapping Ribeye/GluR2 signals in the apical region of female cochlear tissues (control: 15%, CORT: 20%; Mann–Whitney U test, *p* < 0.001). A notable increase was also observed in the medial region of female specimens (from 15% to 18%; Mann–Whitney U test, *p* < 0.001). The overlap of Ribeye/GluR2 signals in male cochlear tissues increased in the middle region (control: 13%, CORT: 15%; Mann–Whitney U test, *p* = 0.002). For all significant changes, Cohen’s effect size was weak (0.1–0.2) (Figure 5).

### 3.6. Baseline Size of GluR2 and Ribeye Puncta

Next, we compared the sizes of GluR2 and Ribeye puncta at baseline. The data distribution was not normal, so we used a non-parametric test for comparison. Although the average size of GluR2 puncta (F: 0.23 ± 0.01, M: 0.26 ± 0.01) was slightly more prominent in male mice, no significant difference was observed between the sexes (Mann–Whitney U test, *p* = 0.845). Similarly, the average size of Ribeye puncta in males (0.29 ± 0.004) did not differ from that in females (0.28 ± 0.004) (Mann–Whitney U test, *p* = 0.828).

### 3.7. CORT Exposure Increases the Size of Ribeye Puncta in Males and Increases the Size of GluR2 Puncta in Female Cochlear Tissues

**Figure 6:**
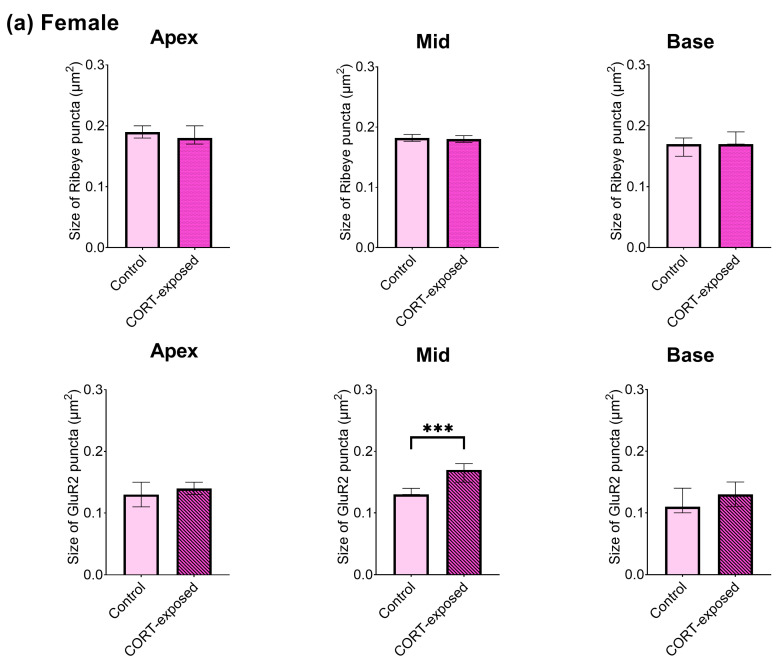

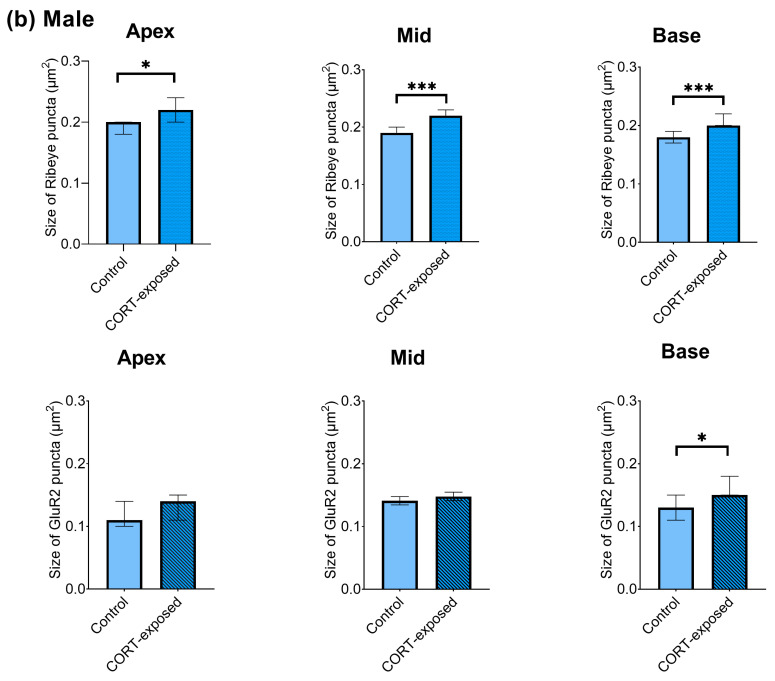
Exposure to CORT influences the size of Ribeye and GluR2 in a sex-dependent manner. The enlargement of GluR2 was observed in females and males, while changes in Ribeye size were seen exclusively in male cochlear explants. (**a**) Significant changes in GluR2 size were observed in the middle part of the cochlea from female mice (control: 0.13 µm^2^, CORT: 0.17 µm^2^ (median values); Mann–Whitney U test, *p* < 0.001) and in the basal region of the cochlea from male mice (control: 0.13 µm^2^; CORT: 0.15 µm^2^ (median values); Mann–Whitney U test, *p* = 0.011); (**b**) changes in Ribeye size in male cochlear explants (an increase of 0.04 µm^2^) occurred throughout the entire cochlear explant (Mann–Whitney U test). For all significant changes, Cohen’s effect size was weak (0.1–0.2). The data are expressed as median values ± 95% CI of median (*n* = 6–8 cochleae for each experimental group, number of analyzed IHCs in total = 2080, average number of analyzed Ribeye/GluR2 puncta in female groups = 6266/2818, average number of analyzed Ribeye/GluR2 puncta in male groups = 5880/3297). Statistical significance is shown in the figure: * *p* < 0.05; *** *p* < 0.001.

Next, we investigated the impact of CORT on the sizes of Ribeye and GluR2 in each sex individually. Exposure to CORT induced significant changes in the size of the Ribeye, which was observed exclusively in male cochlear explants throughout the cochlea. In contrast, an increase in GluR2 size was noted in both male (basal region) and female specimens (middle region) (Figure 6). We compared the changes in shape (width, height, roundness) of both Ribeye and GluR2 puncta. Both male and female cochlear explants showed alterations in the width and height of GluR2, while changes in the shape of Ribeye were observed only in males. To address the general influence of CORT on cochlear morphology, we evaluated intact hair cells and quantified type I and II SGNs, finding them unaffected by CORT exposure (Supplementary Figures S1 and S2).

### 3.8. CORT-Induced Changes in Ribeye Size Are Mediated by the Glucocorticoid Receptor

Glucocorticoids interact with two intracellular receptors: mineralocorticoid (MR) and glucocorticoid (GR). Both MR and GR mediate the genomic and non-genomic effects of CORT. To determine which of these two receptors mediates the CORT function that causes a change in protein composition in the IHC/SGN synapse, the samples were incubated with 100 nM of the GR antagonist mifepristone (MIFE) or 500 nM of the MR antagonist spironolactone (SPIRO) for 20 min, followed by 20 min of incubation with corticosterone and 24 h of culture with tissue culture only. No significant impact of MR and GR antagonists on average GluR2 protein concentration detected by ELISA was observed (MIFE + CORT: 2.48 ± 0.43, SPIRO + CORT: 2.81 ± 0.65, CORT: 2.86 ± 0.66, control: 2.82 ± 0.57).

**Figure 7:**
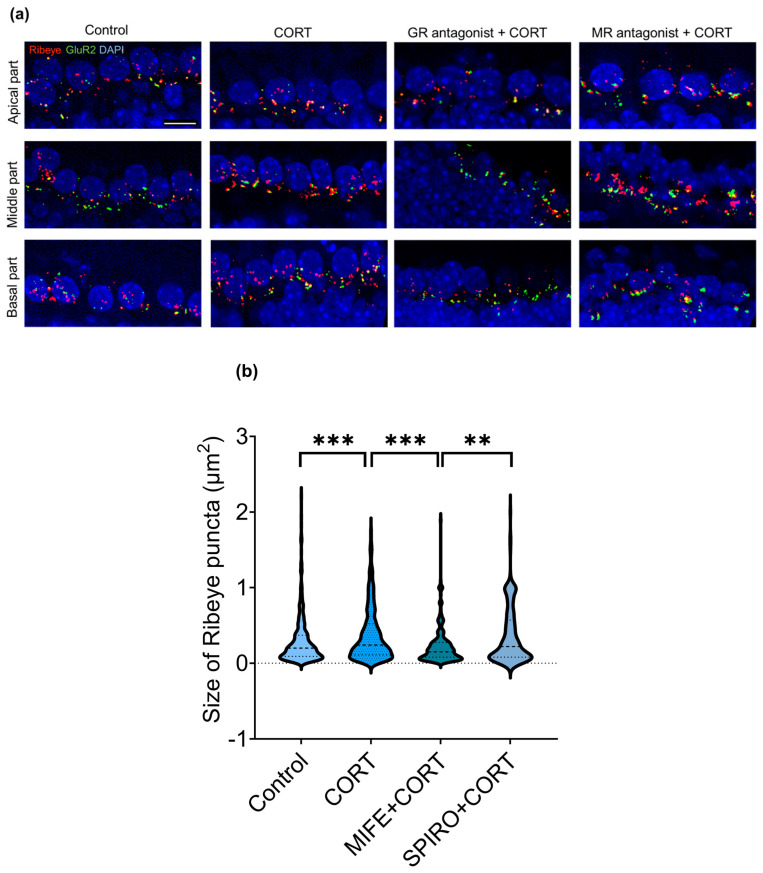
(**a**) Representative confocal images display male cochlear explants with inner hair cell (IHC) synapses in the apex, mid, and base regions. The cochleae were stained with anti-Ribeye (red), anti-GluR2 (green), and DAPI (blue). Scale bar: 10 µm; (**b**) in the middle region of the male cochlea, the Kruskal–Wallis test detected significant differences between CORT and MIFE + CORT groups (CORT: 0.24, MIFE + CORT: 15, *p* < 0.001), but not between CORT and SPIRO + CORT (CORT: 0.24, 0.22, *p* = ns). Median values are presented, with *n* ranging from 2 to 3 cochleae in each experimental group, a total number of analyzed IHCs of 100, and a total number of analyzed Ribeye puncta of 1554. Statistical significance is shown in the figure: ** *p* < 0.01; *** *p* < 0.001.

We then investigated whether the enlargement of Ribeye puncta across the cochlea, caused by CORT in male cochlear explants, could be inhibited by the MR and GR antagonists, SPIRO and MIFE. The Kruskal–Wallis test indicated significant differences between groups (*H*(3) = 37.079, *p* < 0.001). Pairwise comparison revealed differences between the control and CORT group (control: 0.20, CORT: 0.24, *p* = 0.001), MIFE + CORT and CORT group (MIFE + CORT: 0.15, CORT: 0.24, *p* < 0.001), and MIFE + CORT (0.15) and SPIRO + CORT group (0.22, *p* = 0.006). No significant differences were observed between the control and SPIRO + CORT group (*p* = 1.000). Although the Ribeye’s size after incubation with GR antagonist and CORT was smaller, there was no significant difference between the control and MIFE + CORT group (*p* = 0.062) (Figure 7).

## 4. Discussion

This in vitro study examined the impact of short-term corticosterone exposure on the expression and localization of AMPA receptors at inner hair cell synapses in male and female C57BL/6 mice. We observed differences in baseline protein levels of AMPAR subunits between male and female mice, but CORT exposure did not influence their concentrations. Males had more GluR2 puncta on the IHC synapse than females. In contrast, Ribeye puncta showed no such differences (Figure 3). Moreover, CORT exposure increased the size of GluR2 puncta in the middle region of the female cochlea and the basal area for males. In the female but not male cochleae, the number of Ribeye puncta decreased in the middle region after CORT exposure. The size of the Ribeye puncta increased throughout the cochlea but only in males (Figure 6). Furthermore, CORT exposure nearly doubled the colocalized GluR2/Ribeye in the apical region of female cochleae (Figure 4 and Figure 5). Finally, we discovered that glucocorticoid receptor antagonists could block the increase in Ribeye’s size caused by CORT in males (Figure 7).

Our study reveals differences in the basal concentration of GluR2 protein subunits in the cochleae of C57BL/6 P4–P5 pups, with higher levels observed in males than in females. These differences have not been previously addressed in the literature. Existing evidence suggests that female C57BL/6J mice have more Gria2 mRNA particles in the cell bodies of the SGNs located in the basal region of the modiolus than males [29]. However, the animals used in that study and the present one were of different ages (37 days old in the study of Lozier et al. [29], 4–5 days old in our study). Moreover, we measured the protein concentration in the cochlear lysate, whereas Lozier et al. [29] counted the fluorescent puncta in tissue sections, indicating the presence of specific mRNA. This issue needs to be addressed in the future, preferably using both approaches and animals in various developmental stages.

Our study also revealed an intriguing difference between genders regarding the quantity of postsynaptic GluR2 subunits, with no noted variations in Ribeye. Males showed a significantly higher number of GluR2 puncta than females. Changes in the number of Ribeye and AMPARs in C57BL/6 mice during cochlear development have been described in the literature [30,31], but the influence of sex on them remains unreported. Similar to our study, the sex-dependent difference in AMPA receptors was detected in the hippocampus of adult Wistar rats. Males had more AMPA, kainate, and NMDA receptors than females [32]. Observed differences can be related to maturation and sex hormones and should be further investigated. A recent study shows that gonadal hormones can affect the maturation of ABR wave I amplitude, indicating the activity of the auditory nerve in C57BL/6J mice [33]. Future studies should investigate whether sex differences influence the number of GluR2 receptors in the fully developed cochlea.

We observed that CORT exposure affected Ribeye differently based on sex, evident by a size increase found solely in males across the cochlea. To our knowledge, such sex-dependent changes in Ribeye have not been previously reported. However, an increase in the volume of Ribeye was noted in noise-exposed mice [34]. Moreover, the size of presynaptic ribbons has been reported to change during cochlear maturation [30] and aging [35]. Surprisingly, in Ribeye knockout mice, there were only minor changes in the physiology of hair cell synapses and slight hearing impairment [36,37]. Changes in size occurring only in males could possibly be explained by sex hormones, differences in synaptic structures, higher translation rate, or lower turnover protein rate.

Our study demonstrated that CORT exposure decreased the number of Ribeye puncta in the female cochleae in the middle region, a trend not observed in males. Interestingly, we observed that the effects of CORT in female mice were linked to body mass. This suggests that slight variations in cochlear maturation associated with body mass may significantly impact susceptibility to corticosterone in females compared to male animals.

Although we did not detect changes in the protein concentration of AMPAR subunits in CORT-exposed cochleae, we noted differences in the size of GluR2 in both males and females after CORT exposure, specifically in the middle part of the cochlea for females and at the base for males. Our data align with studies on hippocampal neuronal cultures showing that 20 min of exposure to CORT enhances GluR2 surface immunostaining [10,21]. Additionally, 10 min exposure to CORT increases the lateral diffusion of GluR2 [38]. Furthermore, 3 h of CORT exposure led to increased surface expression of GluR1 and GluR2 in hippocampal cultures, with GluR2 demonstrating a more pronounced enhancement and increased surface but not total expression [10]. Such changes were not observed in cultured hippocampal neurons after 15 min of exposure to CORT [39], suggesting the importance of timing in such experiments and indicating a dose- and time-dependent effect of CORT. We established that CORT generally increases GluR2 staining in cochlear explants; however, future studies should explore different concentrations and exposure durations.

We found that the colocalization pattern of GluR2/Ribeye changed following CORT exposure, increasing in the apical and middle regions in female cochleae and the basal region in male cochleae. This suggests that CORT influences cochlear synaptic morphology. Future studies should analyze the pillar and modiolar sides of IHCs separately, as they vary in the size of Ribeye and GluR2 (with more prominent ribbons and smaller GluR2 on the modiolar side compared to smaller ribbons and greater GluR2 on the pillar side at IHCs) [40].

Finally, using the specific inhibitor mifepristone, we found that the increase in size of the Ribeye in male cochlear explants is mediated by glucocorticoid but not mineralocorticoid receptors. Similar to our study, the increase in GluR2 surface staining cultured cells from rat hippocampus exposed to CORT for 20 min was also mediated by glucocorticoid receptors. The observed effect was mimicked by 100 nM of GR agonist RU28362 and was prevented by prior incubation with a selective GR antagonist RU38486 [21]. Mifepristone has been successfully used in the auditory system to attenuate noise-induced hearing loss in female Wistar rats. One of its demonstrated effects is protection from noise-induced loss of inner hair cell ribbons [41].

To date, the impact of stress on the auditory system has been studied in vivo. In male rodents, acute restraint stress protects mice from acoustic trauma [42], whereas chronic restraint stress leads to neuronal atrophy in the inferior colliculus [43]. On the other hand, the hearing abilities of female rodents subjected to subacute stress were strain-dependent, with either worsening or improvement, as measured by ABR and DPOAE [16,44]. Thus, to better understand stress-induced auditory conditions, such as tinnitus and hyperacusis, there is a need to study stress-induced hearing changes separately in both male and female animals.

## 5. Limitations of the Study and Future Directions

The primary limitation of our study was the high intergroup variability, which may be attributed to the animals’ age. The auditory periphery of P4–P5 mice remains immature as they undergo developmental changes until P12, when hearing onset occurs, involving maturation influenced by glutamatergic transmission in the SGNs [45]. GluR1 is also believed to be absent in the mature cochlea [46], while in the central nervous system, GluR1 and GluR2 play a role in AMPAR plasticity and function [47]. Furthermore, studies have shown that gonadal hormones such as estrogen may influence neuroplasticity [47]. The fully mature cochlea would thus likely react differently to CORT exposure, which should be addressed in future research.

Another drawback of this study is the use of an in vitro model. Isolated and cultured primary tissues likely react differently from the organism. The primary cause of this discrepancy is the separation from the vascular and neural systems and the absence of an ionic gradient in the dissected cochlea, both of which are essential for adequate hearing [48]. Nevertheless, in vitro studies help study single effects and optimize in vivo studies [49].

Our study’s third pitfall is focusing only on the HPA axis, whereas stress can also affect the inner ear through the sympathetic–adrenal–medullary axis (SAM). In response to the activation of the SAM axis, the cochlear microvasculature might be affected [50], which cannot be studied using our in vitro model. Another drawback is the use of only one species of animal. Our study was conducted in mice, whereas in vitro studies examining the impact of corticosterone on AMPARs in the central nervous system were predominantly performed in rats [11].

The fifth limitation of our study is that we assessed the changes after CORT exposure only at one time point (24 h) and used only one exposure duration (20 min). This experimental design allows for the possibility that some CORT-induced changes may go unnoticed. Additionally, it eliminates the chance to study the compensatory mechanism.

Considering glutamate’s crucial role in auditory processing, future research should investigate the effects of CORT on other glutamate receptors, such as N-methyl-D-aspartate (NMDA) and kainate receptors. Based on the sex-dependent differences in NMDA receptors [13], future studies should be performed on both males and females. Additionally, studies on the influence of the circadian rhythm on the cochlear stress response should be conducted to understand better the role of a cochlear circadian clock in stress [51]. Finally, assessing the results at various intervals following CORT exposure would yield a more comprehensive understanding of the impact of stress hormones on the AMPA receptor.

## 6. Conclusions

Our study demonstrates that brief exposure to CORT influences the distribution, size, and colocalization of components of the inner ear synapse (Ribeye and GluR2) in a sex-dependent manner, and that glucocorticoid receptors mediate this process. The observed changes align with those occurring during neuroplasticity and provide a foundation for further studies on how stress impacts the auditory periphery in both females and males.

## Figures and Tables

**Table 1:** ELISA kits used to measure the concentrations of AMPA receptor subunits.

Target	Supplier and Catalog Number	Sensitivity (the Minimum Detectable Level)
GluR1	BIOZOL Diagnostica Vertrieb GmbH, Hamburg, Germany, cat. no. ASB-OKEH02153	not provided
GluR2	Hölzel Diagnostika Handels GmbH, Cologne, Germany, cat. no. SEE802Mu-96T	[0.057 ng/mL]
GluR3	Hölzel Diagnostika Handels GmbH, Cologne, Germany, cat. no. SEE803Mu-96T	[0.062 ng/mL]
GluR4	Hölzel Diagnostika Handels GmbH, Cologne, Germany, cat. no. SEE804Mu-96T	[0.054 ng/mL]

**Table 2:** Primary and secondary antibodies and dyes used in the study.

	Target	Antibody Type	Supplier	Catalog No.	Working Dilution
Primary antibodies	Ribeye A-domain	Guinea pig polyclonal	Synaptic Systems GmbH, Göttingen, Germany	192104	1:5000
	GluR2 (clone 6C4)	Mouse monoclonal	Merck Millipore, Darmstadt, Germany	MAB397	1:200
	Anti-neurofilament-200	Mouse monoclonal	Sigma-Aldrich, Taufkirchen, Germany	N0142	1:400
Secondary antibody conjugates	Mouse IgG	Goat anti-mouse Alexa 488 IgG (H + L)	Thermo Fisher Scientific, Darmstadt, Germany	A1100	1:500 1:400
	Guinea pig IgG	Goat anti-guinea pig IgG (H + L) Alexa Fluor 647	Thermo Fisher Scientific, Darmstadt, Germany	A21450	1:500
Other reagents	Prolong Gold DAPI	Mounting solution	Cell Signaling Technology Europe B.V, Frankfurt am Main, Germany	8961S	undiluted
	Phalloidin	iFluor594	Santa Cruz Biotechnology Inc., Heidelberg, Germany	Sc363795	1:1000

**Table 3:** Concentration of AMPAR subunits in the entire membranous cochlea of P4–P5 C57BL/6.

AMPA Subunits	Sex	No. of Samples Included in Statistical Analysis/No. of ELISA Analyzed Samples *	Mean ± SEM	Median	*p*-Value
GluR1	Female	8/10	22.62 ± 3.40	20.44	ns ^1^
	Male	9/10	33.23 ± 4.61	30.26	
GluR2	Female	10/10	2.82 ± 0.18	2.68	<0.001 ^1^
	Male	9/10	6.16 ± 0.63	5.90	
GluR3	Female	4/10	2.48 ± 0.37	2.24	ns ^1^
	Male	6/10	2.99 ± 0.38	3.22	
GluR4	Female	0/10			Not applicable
	Male	2/10	3.75 ± 0.39	3.75	

* Samples with concentrations below the sensitivity limit were excluded from the analysis; ns, not significant; ^1^ data were normally distributed, and a two-tailed unpaired Student’s *t*-test was used.

**Table 4:** The concentration of AMPAR subunits in cochlear tissues dissected from C57BL/6 P4–P5 mice exposed to 20′ CORT and cultured for 24 h.

AMPA Subunits	Sex	Group	No. of Samples Included in Statistical Analysis/No. of ELISA Analyzed Samples *	Mean ± SEM	Median	*p*-Value
GluR1	Female	Control	8/10	22.62 ± 3.40	20.44	ns ^1^
		CORT-exposed	10/10	25.71 ± 3.73	22.03	
	Male	Control	9/10	33.23 ± 4.61	30.26	ns ^2^
		CORT-exposed	9/10	31.37 ± 5.71	24.61	
GluR2	Female	Control	9/10	2.82 ± 0.18	2.68	ns ^2^
		CORT-exposed	10/10	2.86 ± 0.22	2.57	
	Male	Control	9/10	6.16 ± 0.63	5.90	ns ^1^
		CORT-exposed	8/10	5.84 ± 0.43	5.69	
GluR3	Female	Control	4/10	2.48 ± 0.37	2.24	ns ^2^
		CORT-exposed	3/10	2.85 ± 0.21	2.80	
	Male	Control	6/10	2.99 ± 0.38	3.22	ns ^1^
		CORT-exposed	6/10	2.79 ± 0.26	2.66	
GluR4	Female	Control	0/10			Not applicable
		CORT-exposed	0/10			
	Male	Control	2/10	3.75 ± 0.39	3.75	Not applicable
		CORT-exposed	4/10	3.62 ± 0.55	3.61	
		CORT-exposed	0/10			

* Samples with concentrations below the sensitivity limit were excluded from the analysis; ns, not significant; ^1^ data were normally distributed, a two-tailed unpaired Student’s *t*-test was used; ^2^ distribution of data was not normal, the non-parametric Mann–Whitney U test was used.

## Data Availability

The raw data supporting the conclusions of this article will be made available by the authors on request.

## Supplementary Materials

The following supporting information can be downloaded at https://www.mdpi.com/article/10.3390/brainsci15050441/s1: Figure S1: A. Exposure to 100 nM corticosterone (CORT) does not affect cochlear morphology. Representative epifluorescence images of hair cell stereocilia bundles were visualized using phalloidin-iFluor-594 labeling. Cells with non-disrupted stereocilia were defined as intact HC. The micrograph represents the medial part of the cochlea exposed to CORT pulse for 20 min. The graph plots the mean percentage of intact hair cells. Using a *t*-test, we excluded the effect of CORT on IHC (t(6) = −0.413, *p* = 0.694) and OHCs (t(6) = −0.019, *p* = 0.985), with *n* = 4 per group. Mean ± SEM are reported. B. The number of neurites did not differ between the control group (*n* = 4) and the CORT-exposed group (*n* = 4) (t(18) = 0.07, *p* = 0.945). The explants were labeled with NF200 and a secondary antibody conjugated to Alexa Fluor 488. The areas containing the SGN peripheral axons were quantified in 10 representative sections. The images represent the basal part of the cochlea in the control group (B1) and the corticosterone-exposed group (B2). The data represents three independent experiments. Means ± SEM are reported; Figure S2: Exposure to 100 nM CORT does not impact the turning of SGN Type II; Figure S3: Body mass [g] of C57Bl/6 mice (P4–P5) included in the immunofluorescence study on IHC synapses (IF against Ribeye, GluR2); Table S1: The ΔCt and ΔΔCt values of GRIA2 gene expression throughout the membranous cochlea at 3 or 6 h after acute exposure to CORT.

## Abbreviations

The following abbreviations are used in this manuscript:

AMPA	α-amino-3-hydroxy-5-methyl-4-isoxazolepropionic acid
AMPAR	the ionotropic glutamate receptor AMPA
CORT	corticosterone
GluR1	AMPA receptor GluR1 subunit
GluR2	AMPA receptor GluR2 subunit
GluR3	AMPA receptor GluR3 subunit
GluR4	AMPA receptor GluR4 subunit
GR	glucocorticoid receptor
IHCs	inner hair cells
MR	mineralocorticoid receptor
SGNs	spiral ganglion neurons
