# Supplementary material for: The Size and Localization of Ribeye and GluR2 in the Auditory Inner Hair Cell Synapse of C57BL/6 Mice Are Affected by Short-Pulse Corticosterone in a Sex-Dependent Manner
Source: Brain Sci. 2025 Apr 24;15(5):441. doi: 10.3390/brainsci15050441 (PMC12110336; doi:10.3390/brainsci15050441)
Supplement: Supplementary file 1 [file brainsci-15-00441-s001.zip › brainsci-3579746-supplementary.pdf]

Supplementary Figure S1

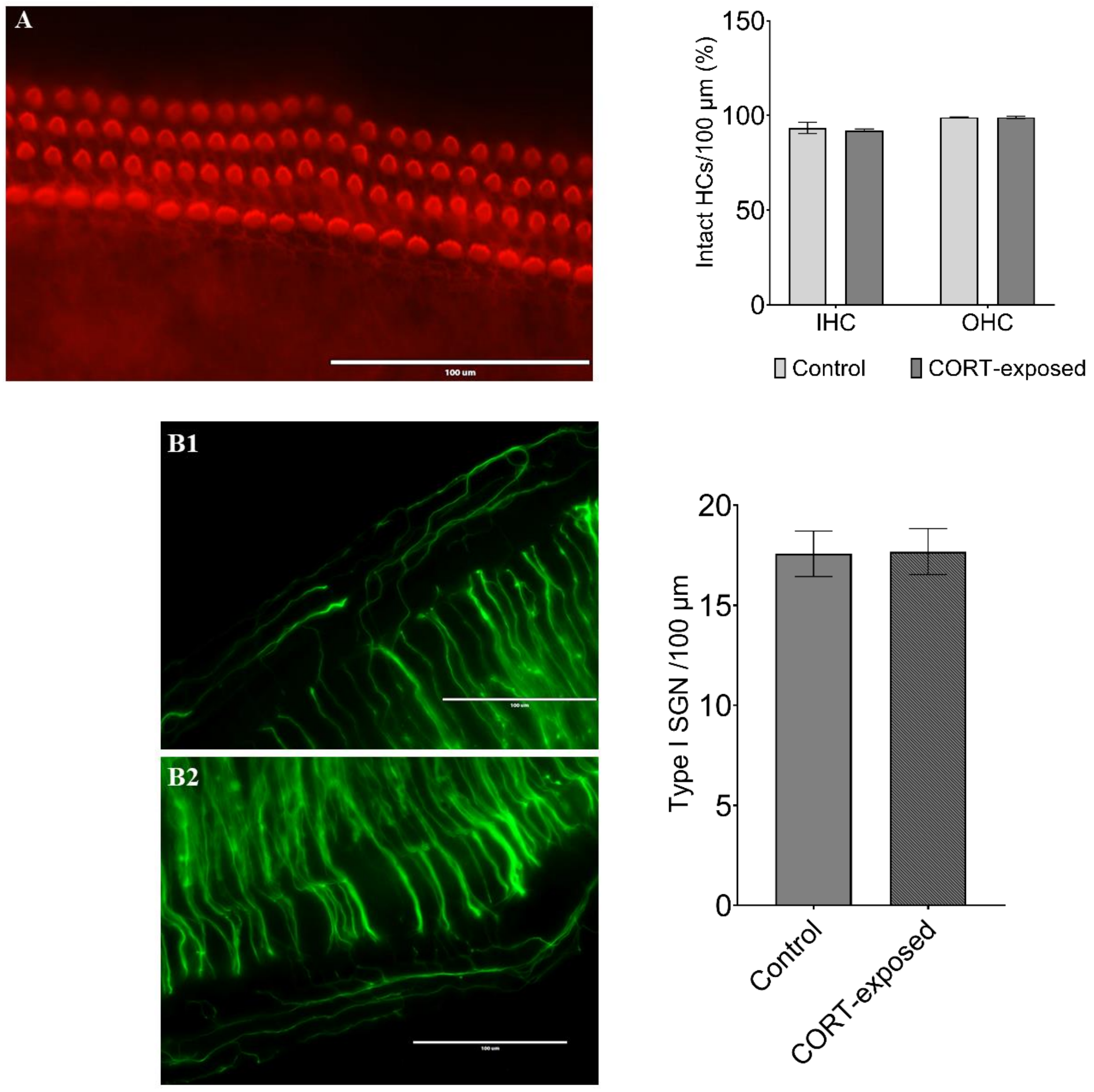

Supplementary Figure S1. **A**. Exposure to 100 nM CORT does not affect the cochlear morphology. The representative epifluorescence images of hair cell stereocilia bundles were visualized by phalloidin-iFluor-594 labeling. Cells with a non-disrupted stereocilia were defined as intact HC. The microphraph represents the medial part of the cochlea exposed to CORT pulse for 20 minutes. The graph plots the mean percentage of intact hair cells. Using a t-test, we excluded the effect of CORT on IHC ($t(6)= -0.413$, $p=0.694$) and OHCs ($t(6)=-0.019$, $p=0.985$), $n=4$ per each group. Mean ± SEM are reported. **B**. The number of neurites did not differ between the control ($n=4$) and the CORT-exposed group ($n=4$) ($t(18)=0.07$, $p=0.945$). The explants were labeled with NF200 and a secondary antibody conjugated with the Alexa FluorTM 488. The areas containing the SGN peripheral axons were quantified in 10 representative sections. The images represent the basal part of the cochlea in the control group (B1) and the corticosterone-exposed group (B2). The data represents three independent experiments. Means ± SEM are reported

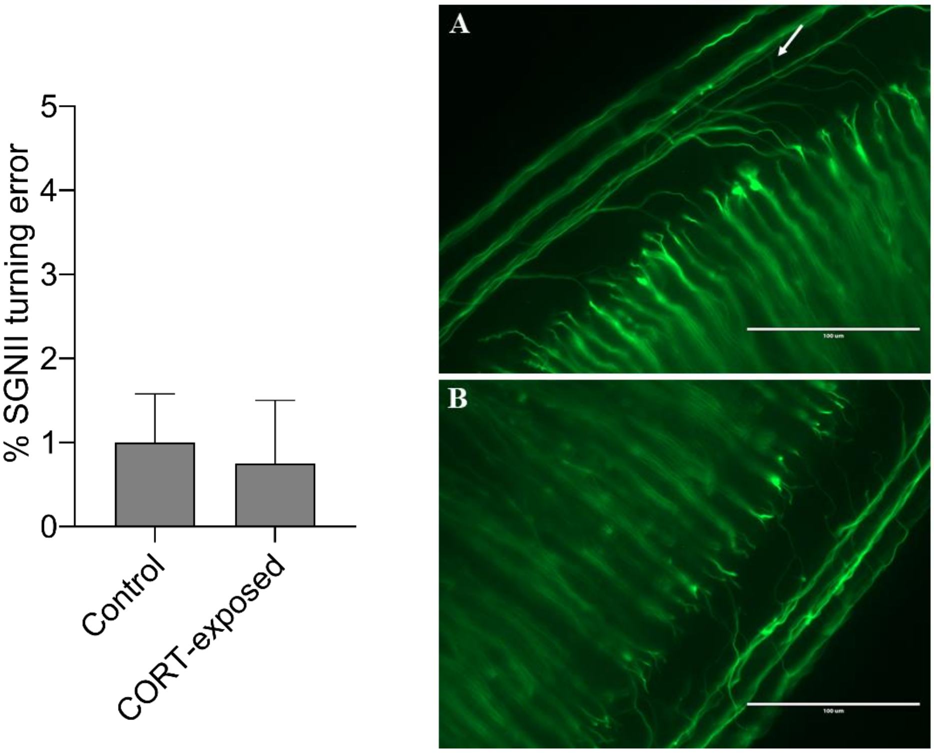

Supplementary Figure S2. Exposure to 100 nM CORT does not impact on turning of SGN Type II. No differences between CORT-exposed and control group in SGN Type II were detected (t(6)=-0.264, p=0.801). The image represents the medial part of the cochlea exposed to CORT (A) and control (B). SGN Type II were quantified according to the instruction developed by Clancy et al. [28] Arrow indicates the example of incorrect turning SGN Type II. Using the t-test we have excluded the impact of corticosterone on SGN Type II (n=4 per each group). Means ± SEM are presented.

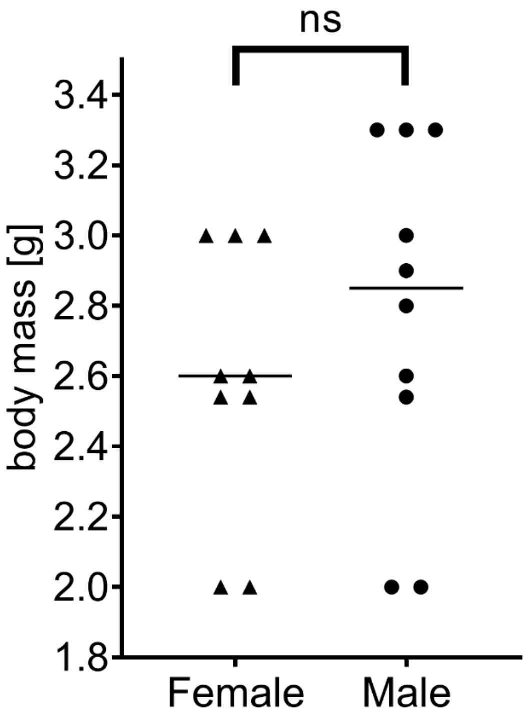

Supplementary Figure S3. Body mass [g] of C57Bl/6 mice (P4-5) included in the immunofluorescence study on IHC synapses (IF against Ribeye, GluR2). Median value of body mass in female group was 2.6. whereas in male 2.9.

Supplemtary Table S1. The ΔCt and ΔΔCt values of GRIA2 gene expression throughout the membranous cochlea at 3 or 6 hours after acute exposure to CORT. The hippocampus and spleen served as positive and negative controls.

Time point			Sample	Ct ßeta-Actin (Duplicate 1)	Ct ßeta-Actin (Duplicate 2)	Avarage Ct (ßeta-Actin)	Ct GRIA2 (Duplicate 1)	Ct GRIA2 (Duplicate 2)	Avarage Ct (GRIA2)	ΔCt (Average Ct GRIA2-Average Ct ß-Actin)	ΔΔCt	2^-(ΔΔCt)
3 hours	male	Control	cochlea 1	18.53	18.52	18.53	25.65	25.49	25.57	7.05	-0.04	1.0
			cochlea 2	17.83	17.71	17.77	24.97	24.83	24.90	7.13	0.04	1.0
		CORT exposed	cochlea 1	17.79	17.73	17.76	25.74	25.84	25.79	8.03	0.94	0.5
			cochlea 2	18.53	18.40	18.47	25.47	25.42	25.45	6.98	-0.11	1.1
			cochlea 3	18.61	18.36	18.49	25.09	25.13	25.11	6.63	-0.46	1.4
	female	Control	cochlea 1	18.08	18.14	18.11	24.68	24.72	24.70	6.59	-0,25	1.2
			cochlea 2	18.93	19.09	19.01	26.11	26.07	26.09	7.08	0.25	0.8
		CORT exposed	cochlea 1	17.99	18.09	18.04	24.79	24.79	24.79	6.75	-0.09	1.1
			cochlea 2	18.80	18.76	18.78	25.76	25.63	25.70	6.92	0.08	0.9
			cochlea 3	17.70	17.69	17.70	26.01	25.82	25.82	8.13	1.29	0.4
6 hours	male	Control	cochlea 1	17.00	16.92	17.00	24.66	24.61	24.64	7.64	0.19	0.9
			cochlea 2	17.08	17.24	17.16	24.39	24.45	24.42	7.26	-0.19	1.1
		CORT exposed	cochlea 1	16.97	17.20	17.09	25.18	24.83	25.01	7.92	0.47	0.7
			cochlea 2	16.29	16.54	16.42	23.90	23.95	23.93	7.51	0.06	1.0
	female	Control	cochlea 1	17.42	17.47	17.45	24.88	24.73	24.81	7.36	0.01	1.0
			cochlea 2	16.90	16.66	16.78	24.13	24.11	24.12	7.34	-0.01	1.0
		CORT exposed	cochlea 1	17.27	17.22	17.25	24.41	24.38	24.40	7.15	-0.20	1.1
			cochlea 2	16.72	16.92	16.82	24.43	24.45	24.44	7.62	0.27	0.8
		Negative Control	spleen 1	14.70	14.82	14.76	27.38	27.65	27.52	12.76	5.36	0
			spleen 2	14.55	14.55	14.55	27.67	27.54	27.61	13.06	5.66	0
		Positive Control	hippocampus 1	15.20	15.05	15.13	16.78	16.83	16.81	1.68	-5.55	35.6
			hippocampus 2	15.29	15.28	15.29	16.92	16.88	16.90	1.62	-5.22	37.3
